# Indicators of food and water security in an Arctic Health context – results from an international workshop discussion

**DOI:** 10.3402/ijch.v72i0.21530

**Published:** 2013-08-07

**Authors:** Lena Maria Nilsson, James Berner, Alexey A. Dudarev, Gert Mulvad, Jon Øyvind Odland, Alan Parkinson, Arja Rautio, Constantine Tikhonov, Birgitta Evengård

**Affiliations:** 1 Arctic Research Centre, Umeå University, Umeå, Sweden; 2 Department of Public Health and Clinical Medicine, Nutritional Research, Umeå University, Umeå, Sweden; 3 Alaska Native Tribal Health Consortium, Anchorage, AK, USA; 4 Northwest Public Health Research Center, St-Petersburg, Russia; 5 Greenland Center for Health Research, University of Greenland, Nuuk, Greenland; 6 Faculty of Health Sciences, University of Tromsø, Tromsø, Norway; 7 Arctic Investigations Program, US Centers for Disease Control and Prevention, Anchorage, AK, USA; 8 Thule Institute, University of Oulu, Oulu, Finland; 9 Environmental Public Health Division, First Nations and Inuit Health, Health Canada, Ottawa, Canada; 10 Division of Infectious Diseases, Department of Clinical Microbiology, Umeå University, Umeå, Sweden

**Keywords:** food security, water security, indicators, Arctic people, public health, climate change

## Abstract

In August 2012, a literature search with the aim of describing indicators on food and water security in an Arctic health context was initialized in collaboration between the Arctic Human Health Expert Group, SDWG/AHHEG and the AMAP (Arctic Monitoring and Assessment Programme within the Arctic Council) Human Health Assessment Group, AMAP/HHAG. In December 2012, workshop discussions were performed with representatives from both of these organizations, including 7 Arctic countries. The aim of this article is to describe the workshop discussions and the rational for the 12 indicators selected and the 9 rejected and to discuss the potential feasibility of these. Advantages and disadvantages of candidate indicators were listed. Informative value and costs for collecting were estimated separately on a 3-level scale: low, medium and high. Based on these reviews, the final selection of promoted and rejected indicators was performed and summarized in tables. Among 10 suggested indicators of food security, 6 were promoted: healthy weight, traditional food proportion in diet, monetary food costs, non-monetary food accessibility, food-borne diseases and food-related contaminants. Four were rejected: per-person dietary energy supply, food security modules, self-estimated food safety and healthy eating. Among 10 suggested indicators of water security, 6 were promoted: per-capita renewable water, accessibility of running water, waterborne diseases, drinking-water-related contaminants, authorized water quality assurance and water safety plans. Four were rejected: water consumption, types of water sources, periodic water shortages and household water costs.

To monitor food and water security is an effort in many different parts of the world, not least in developing countries of southern latitudes struggling against acute starvation, malnutrition and thirst (1). In northern latitudes, food and water security appear in a less obvious way. Consequently, little is known about efforts needed to achieve a situation when all Arctic people (indigenous and non-indigenous) at all times have access to sufficient, safe, nutritious food and water to maintain a healthy and active life (2).

The Arctic environment is in a state of change due to global warming, pollution and is of increasing economic interest for the region. Food and water security are highly affected by all these drivers (3–5). Consequently, during the Swedish chairmanship of the Arctic Council 2011–2013, food and water security was highlighted as a priority issue. A joint project was initiated, aiming at providing a “basis for indicator selection that is relevant for food and water security in the Circumpolar areas and which could be used in international collaborations of surveillance in the Arctic” (2).

A large number of indicators of food and water security have previously been used, both within and outside an Arctic health context (1,6,7). Thus, in August 2012, a general search on the literature published from January 2000 to September 2012, as well as a search in official open-source databases related to food and water security issues was performed with a focus on measures already described and used in an Arctic health context (2). The goal was to identify universal and informative summary measures to demonstrate temporal changes in food and water security in the Arctic population, according to established monitoring methodology (8), and the indicators based on individual measures should be presented in gender-divided tables, since climate change affects men and women differently (9,10). It was also stated that availability of traditional food would require special attention, not least because of its high importance from an indigenous perspective (11–16).

The aim of this article is to describe the indicators on food and water security that were discussed during this workshop with experts from 7 Arctic countries and the rational for the 12 indicators selected and the 9 rejected, as well as the potential feasibility of the former.

## Alternative measures considered by the workshop

A total of 20 candidate indicators of food and water security based on the literature and database search were sorted and discussed according to availability, accessibility and safety as summarized in Table I. Food and water availability was defined as a situation when a sufficient quantity of food and water is available on a consistent basis (17). Food and water accessibility was defined as having sufficient resources to obtain appropriate foods for a nutritious diet (17) and water for drinking and hygienic needs. Food and water safety was defined as prevention of illness, disability and death due to food- and waterborne diseases, both infectious and non-communicable, such as poisoning by contaminants or unhealthy eating. In some cases, more than one aspect of food and water security was covered by the same candidate indicator. Therefore, sorting contributed to a process of weighting which aspect to consider as the most significant.

**Table I:** Candidate indicators of food security in an Arctic health context sorted according to security aspect reflected by the measure and the general workshop discernment

Security aspect	Candidate Indicators	Number of alternative measures considered	Number of indicators discerned by workshop to …			Promoted measure

			promote	develop	reject	
Food availability	Healthy weight	3	2	0	1	BMI (kg/m^2^) Percentage obese (BMI >30 in adults, or>+ 2 SD in children)
	Traditional food proportion in diet	3	2	0	1	Self-estimated % traditional food in diet Proportion eating TF the previous day or week
	Per-person dietary energy supply	1	0	New	1	–
Food accessibility	Monetary food costs	3	1	0	2	Cost for nutritious food basket, percentage of disposable household income
	Non-monetary food accessibility	5	1	4	0	Percentage of families/households with hunter/fisher/ collector/herder
	Food security survey modules	2	0	1	1	–
Food safety	Food-borne diseases	3	3	0	0	Incidence rate in humans, Seroprevalence in human, Seroprevalence in subsistence species
	Food-related contaminants	3	3	0	0	Chemical contaminants in food, Microbiological contaminants in food, Chemical contaminants in human tissue
	Self-estimated food safety	1	0	0	1	–
	Healthy eating	3	0	1	2	–
SUM		27	11	6+new	10	N=11 measures

Advantages and disadvantages of candidate indicators were listed. Informative value and costs for data collection were estimated separately on a 3-level scale: low, medium and high. Based on these reviews, the final selection of promoted and rejected indicators was performed.

## Ethical considerations

The results of these workshop discussions have previously been published in a non-peer-reviewed report (2). In order to stimulate a broader scientific dialogue on feasible indicators of food and water security in an Arctic health context, we find it necessary to restructure the presentation of the discussion to make our conclusions more easily available for review by other scientists in public health.

## Indicators discerned by the workshop to promote, develop or reject

In the following sections, candidate indicators and the result of the workshop discussions are summarized according to food and water security aspects and final results, as presented in Tables I and II.

**Table II:** Candidate indicators of water security in an Arctic health context sorted according to security aspect reflected by the measure and the general workshop discernment

Security aspect	Candidate Indicators	Number of alternative measures considered	Number of indicators discerned by workshop to…			Promoted measure

			promote	develop	reject	
Water availability	Per-capita renewable water	1	1	0	0	(m^3^/capita and year)
	Water consumption	1	0	0	1	–
	Types of water sources	4	0	new	4	–
Water accessibility	Accessibility of running water	5	1	0	4	Percentage of households having running water available in their homes
	Periodic water shortages	2	0	2	0	–
	Household water costs	1	0	0	1	–
Water safety	Waterborne diseases	1	1	0	0	Incidence rate in human
	Drinking-water-related contaminants	3	3	0	0	Exceedings of national threshold levels, Occasions when consumers have been recommended to boil their drinking water, Microbiological quality of water
	Authorized water quality assurance	1	1	0	0	Proportion of consumers having access of authorized quality assured water
	Water safety plans	1	1	0	0	Presence of a compulsory water safety plan according to WHO
SUM		20	8	2 +new	10	

### Healthy weight

Three alternative measures of healthy weight were discussed, namely body mass index (BMI) (kg/m^2^), proportion underweight (%) and proportion obese (%).

Healthy weight reflects the actual energy balance of an individual (energy balance=energy intake−energy expenditure). A high proportion of underweight persons in a population is an indicator of insufficient availability of food on a population level and a high proportion of obese people reflects prevalent unhealthy food choices.

On an individual level, BMI has been questioned as a measure of overweight and obesity, since people who are very athletic may have a BMI that is higher than recommended levels. Similarly, from an underweight perspective, a low BMI may have other causes than the insufficient availability of food, such as diseases affecting the metabolism (18). BMI was promoted as an indicator because it is a universally used measure (15,16,19–22), it is easy to calculate and is likely abundantly collected at least in children all over the Circumpolar area. Both obesity and BMI were considered measures with a relatively high information value to the relatively low cost of collection. Obesity was pointed out as an alternative measure, if, e.g. some areas use alternative measures of metabolic imbalances (e.g. skin-fold measures). Underweight was not promoted as an indicator of food security in an Arctic health context, since being underweight was not considered to be a general problem.

### Traditional food proportion in diet

Three different measures of traditional food proportion in diet were discussed, namely: energy percentage of traditional food in diet (20,23), proportion of the population that consumed traditional food the previous day (24) and self-estimated proportion of traditional food in diet (25). A fourth alternative measure based on biomarkers, such as stable nitrogen and carbon isotope ratios, was excluded from the discussion because of limited Circumpolar experience of this method (26).

The proportion of traditional food in the diet was considered a useful indicator of traditional food security. All measures of traditional food proportion in diet were considered to have a moderate information value from a food security perspective and data collection cost was estimated to be relatively high. Despite this, the workshop promoted surveying this indicator, since this indicator also could serve as a proxy indicator of cultural strength. It was also stressed that a single measure of traditional food in diet the previous day may be biased. The measures promoted were thus the proportion eating traditional food the previous day and week and the self-estimated proportion of traditional food in diet.

### Per-person dietary energy supply

Per-person dietary energy supply (available kcal/person/day) is a commonly used indicator for food security (1). It is defined as food available for human consumption within a certain area or population. From an individual health aspect, there is an overestimate built into this measure, since some food might be wasted. Furthermore, the amount of household waste has been demonstrated to increase disproportionately strongly with rising levels of available energy (27).

Per-person dietary energy supply was not considered to be a useful indicator of food security in the Arctic area, since this area is generally food sufficient, because of food availability support programs such as food banks and community freezers. Both information value and monitoring cost were estimated to be low.

As an alternative measure, a new indicator: food availability support programs, such as food banks and community freezers, was suggested. Do food availability support programs exist? And, if they exist, on which level are they active (society, region, community, charity)?

### Monetary food costs

Three alternative measures of monetary food costs were discussed, namely the cost of a general food basket (28), the cost of a nutritious food basket (29), and the cost of a 4-week healthy menu (30). To make costs comparable on an international level, with different currencies involved, all measures were supposed to be presented as a proportion of disposable household income.

Among suggested measures, a standardized nutritious food basket was promoted, a method that has been used in Canada since 1975 to monitor the cost and affordability of healthy eating (29). This food basket has been deemed a practical proxy estimate of individual food accessibility, since it can be implemented easily and quickly at low cost (31). Though no directly comparable measures have been collected in different communities, this local monitoring has shown that food costs in the Canadian north may be more than twice as high as in the south (32,33).

The cost of a nutritious food basket was considered an indicator of high information value with a low monitoring cost, and a comparable, practical and potentially universal/standardized measure. However, it was stressed that this indicator needs to be correlated to indicators on purchasing power, that it may not be reflective of actual consumption, and that it is not yet standardized for use all over the Circumpolar area. Furthermore, the validity of measuring the cost for a healthy food basket in countries such as Russia was questioned, since food costs have fluctuated tremendously during the last decade with different magnitudes in different regions.

### Non-monetary food accessibility

Five alternative measures of non-monetary food accessibility were discussed, namely the presence of hunter/fisher/collector/herder in families/households (34), accessibility of hunting/fishing/collecting/herding equipment, accessibility of sufficient hunting/fishing/collecting/herding land areas, environmental conditions suitable for hunting/fishing/collecting/herding, and coping strategies to obtain traditional foods (25).

In most countries no single agency surveys all 5 suggested measures, and none of the measures are surveyed regularly. In Arctic literature, only measures of the presence of a hunter in the family, and coping strategies to achieve traditional foods are apparent from an abstract review perspective (25,34). Among the suggested measures, the presence of hunter/fisher/collector/herder in the family was considered the easiest to monitor, and thus the most feasible. However, none of the other suggested measures were considered impossible to develop into a relevant survey measure in the future. All indicators describing conditions for hunting/fishing/collecting/herding was considered potentially relevant, with all the advantages and disadvantages of survey data. It was also pointed out that repeated surveys of a representative subpopulation could be useful for showing trends. The suggested measure presence of hunter/fisher/collector/herder in the family was considered an indicator of medium information value to a medium monitoring cost.

Concerning coping strategies to achieve traditional foods, as measured within the SLiCA (A Survey of Living Conditions in the Arctic) survey, 4 questions covering harvesting traditional food, getting traditional food in exchange for assisting others, getting traditional food in exchange for other food and receiving traditional food as a gift, have previously been used (25). Coping strategies was considered an important indicator from an indigenous perspective, though presently it has not been collected longitudinally. The information value was considered high and the estimated cost for monitoring was considered medium to high. It was discussed whether or not some of the 4 measures of the SLiCA questionnaire could be merged. A new measure, received from society or community (e.g. food banks), was also suggested.

### Food security survey modules

Two alternative measures of food security survey modules (FSSMs) were discussed, namely the number of affirmative answers (0–18) in the US Department of Agriculture (USDA) FSSM (35) or a Canadian version adapted to the northern indigenous population (36), respectively. FSSMs can be seen as methods of combining different food security indicators, focusing on the actual consumption narrative of the individual/family, that is, the narrative of food accessibility, into one measure. The score is calculated by the number of affirmative answers in the questionnaire, 10 questions for adults and 8 questions for children. In North America, FSSM is a well-established survey method, which could be a useful indicator if repeated regularly in the same population. However, since this indicator was considered a medium information value to a relatively high cost, and not universally used over the entire Circumpolar area, it was considered a less valuable indicator to promote. Thus, among the suggested measures, both the adapted Canadian FSSM version and the USDA questionnaire were deemed to be in need of further development.

### Food-borne diseases

Three alternative measures of food-borne diseases were discussed, namely the incidence rate in humans, seroprevalence in humans, and seroprevalence in subsistence species. It was stated that public health surveillance data on food-borne diseases is collected continuously in many countries and is thus a widely available indicator, and all suggested measures were promoted.

Regarding the incidence rate of food-borne diseases in humans, it was stressed that many cases may be underdiagnosed. Thus, it is rather an indirect than a direct measure of how safe food is. However, this was the only possible indicator to use for comparisons with Russia. The measure of seroprevalence of food-borne diseases in humans tells us about the population at risk. It is an indicator of high predictive value.

By measuring seroprevalence, underdiagnosed cases will also be identified, and already collected biological samples may be used for this monitoring. A disadvantage of this indicator is that there may be a limited availability of human biological samples in some countries, and there is a need to develop universal methods for the area, to make the results comparable. The information value is high, but monitoring costs may be medium to high both regarding incidence rate and seroprevalence in humans.

Seroprevalence in subsistence species is an important issue to follow, for information to the population. However, it is a high cost for collecting this information with data not being available in all countries.

### Food-related contaminants

Three alternative measures of food-related contaminants were discussed, namely, chemical contaminants in food, microbiological contaminants in food and chemical contaminants in human tissue. As data are collected continuously in many countries, this is a widely available indicator of food security.

Despite high monitoring costs, all suggested measures were promoted because of their high information value, and it was stressed that it is important to collaborate with the Arctic Monitoring and Assessment Programme within the Arctic Council's Human Health Assessments Group (AMAP/HHAG) which already supports biomonitoring of food-related contaminants in the Arctic (5,37–39).

Regarding chemical contaminants in food, it was stressed that fish, marine mammals, reindeer and caribou are of particular interest from an Arctic health perspective. It was also stressed that it is important to monitor all kinds of food, not only traditional food. Chemical contaminant data from humans may be limited in Russia.

Regarding food-related contaminants, concentrations as well as exceedances of threshold levels are usually monitored. The workshop stated that exceeding threshold levels is an over-simplification. Thus, a more complete data presentation was warranted.

### Self-estimated food safety

One measure, previously used in the SLiCA survey (25), was suggested as a candidate indicator of self-estimated food safety (question H10d). However, the value of this kind of self-reported risk assessment may be questioned, since people may be more or less afraid of environmental threats than evidence warrants (40). Self-estimated food safety was considered as easy to collect, but of limited value since it is too complicated an issue for people to be able to report adequately. As both information value and monitoring cost were estimated to be low, it was rejected by the workshop.

### Healthy eating

Among the 3 suggested indicators of healthy eating, none were promoted. The consumption of fruit and vegetable indicator was deemed to be potentially feasible but in need of further development, and the other two, healthy eating index and macronutrient distribution, were not supported. The difficulty in defining what is healthy and what is not healthy with a cultural perspective was stressed. However, data on fruit and vegetable consumption as a proxy for healthy eating was found to be regularly monitored and available in many Arctic countries. Thus, it was stated that this measure could add a value for policy makers. It was discussed whether fruit and vegetable consumption should be merged or be presented as two separate measures. The informative value was considered relatively low, and the cost for monitoring medium to high.

### Per-capita renewable water

Per-capita renewable water, measured as m^3^/capita/year, is an estimate of the number of people who can live reasonably with a certain unit of water resources (41). It was promoted as an indicator of water availability, since it was considered to have a high information value and a low estimated monitoring cost, it may be affected by climate change and it is easy to find available data (World Bank, Organization for Economic Co-operation and Development, OECD). However, per-capita renewable water has the disadvantage of ignoring seasonal variability of water available for consumption and the potential for water recycling. Moreover, in large countries with strong regional variations, the mean per-capita renewable water should be presented by region rather than as a country-based average (41).

### Water consumption

Water consumption, measured in litres/person/day, was rejected as an indicator, since there are many difficulties in achieving an accurate measure. Generally, available data only cover centralized water consumption. In some countries, region-specific data are absent. The workshop agreed that this indicator represented a low information value to a high cost.

### Types of water sources

Four measures were suggested to monitor water sources, namely groundwater, groundwater affected by surface water, surface water and other sources. There was a general agreement that none of these suggested measures were good enough in the present state, though types of water sources ought to be monitored, since different sources of water may be differently vulnerable for climate change and pollution. Final categories of water sources should be selected to fit the entire Arctic region.

### Accessibility of running water

Among 5 suggested measures to monitor, the accessibility of running water proportion of households having running water available in their homes was promoted, since it was considered a widely used measure, with a high information value to a low monitoring cost. The other 4 measures, proportion of households having hot running water, cold running water, indoor flushing toilet, or bath or shower, had all been used in the SLiCA study, and were thus previously monitored among indigenous people all over the Arctic. However, since they were not considered to add much information in comparison with only a general measure, they were excluded.

### Periodic water shortages

Two measures were suggested to monitor periodic water shortages, namely number of communities with reoccurring drinking water safety advisories, and occurrence of safety advisories within a certain time (person/days/years). None of these measures were promoted. The workshop concluded that periodic water shortage is a problematic area that might increase in the future, and thus it is important to measure it. However, there was no consensus as to which of the suggested measures was the best fit. Generally, it was thought that a relatively high information value could be retrieved regarding this issue to a relatively low cost.

### Household water costs

Household water cost, measured as a proportion of disposable income, was rejected as an indicator of water security. It was generally considered that data on household water costs do not add much information because of the large variation in cost and considerable seasonal variations. It was also considered to be difficult to achieve comparable data from different countries. Thus, a low information value would be achieved at a high cost.

### Waterborne diseases

Incidence rate of waterborne diseases in humans, measured as cases/100,000 persons/year, was promoted as an indicator for water security. Waterborne infectious diseases have been reported from many Arctic countries, and it has been suggested that changes in the climate will increase the occurrence of these diseases (42,43). As legislation concerning which waterborne diseases that are compulsory to register may vary between different countries, aggregated as well as country-specific Circumpolar data should be presented. Similarly with food-borne diseases, data on waterborne diseases are collected continuously in many countries and are thus a widely available indicator of water security, though many cases may be under-diagnosed. A relatively high information value could be achieved to a relatively low monitoring cost.

### Drinking-water-related contaminants

All suggested measures of drinking-water-related contaminants were promoted, namely exceedances of national threshold levels, occasions when consumers have been advised to boil their drinking water, and microbiological quality of water. The workshop concluded that every country in the Arctic region is already monitoring water-related contaminants, and since all exceedances do not lead to advisories, both numbers of exceedances and proportional values of advisories are of importance to monitor. When measuring contaminants, it is important to stress the fact that threshold levels may differ between the countries.

### Authorized water quality assurance

The suggested measure proportion of the population or households who has access to water sources within the authorities’ water quality control was promoted. It was stressed that there may be difficulties in finding data for people outside authorized water distribution systems. Furthermore, people may have more than 1 house/settlement, and there is a risk that there are systems in different areas of the Arctic that are too diverse to make it suitable for international comparisons. Despite this, a relatively high information value was considered to be possible to achieve at a relatively low cost.

### Water safety plans

The measure indicating the presence of compulsory water safety plan according to WHO (yes/no) was promoted. Water Safety Plans have been launched by the WHO as a way to “ensure safe drinking-water through good water supply practice” (44). The work focuses on securing the entire chain from raw water to the pipes and includes related concepts such as the risk assessment tool HACCP (Hazard Analysis and Critical Control Points) and raw water protection. The presence of water safety plans was considered to be of importance to monitor, though further discussions on this issue are warranted. Similarly with authorized water quality assurance, a relatively high information value was considered to be possible to achieve to a relatively low cost.

## Further use of promoted indicators

The aim of the workshop was to identify universal, accessible and informative summary measures to demonstrate temporal changes in food and water security in the Arctic population, according to the established monitoring methodology. Out of 20 candidate indicators of food and water security, with 47 corresponding measures, 12 indicators were promoted as currently feasible for further initiatives in an Arctic health context. For 5 of these indicators, more than one accurate measure was promoted, and the total number of promoted measures amounted to 19, as shown in Tables I and II. Two of the promoted indicators, that is, traditional food proportion in diet, and non-monetary food accessibility, were selected with consideration of the indigenous population of the Arctic area.

Most of the indicators promoted are already regularly monitored or surveyed in many of the Arctic countries. However, they are often used in a context other than food and water security, and only indicators related to environmental contaminants have so far been systematically monitored in Arctic joint actions (5,37–39).

One strength in the indicator selection was the broad anchoring of the project. The work was performed in collaboration between the Arctic Human Health Expert Group, SDWG (The Sustainable Development Working Group within the Arctic Council)/AHHEG [The Arctic Human Health Experts Group within the Arctic Council (connected to SDWG)] and the AMAP Human Health Assessments Group, AMAP/HHAG, with representatives from both organizations. Experts from all Arctic countries except Iceland were included in the expert group.

A weakness in the indicator selection was the limited time frames within which the literature search and workshop discussions were performed, from August 2012 to February 2013. Given that further discussions had been possible, some indicators deemed to need further development, might have been included among promoted indicators, and more or fewer measures may have been promoted or rejected.

However, to get a more complete vision of the feasibility of selected indicators, they should not just be discussed. There is a widespread awareness that climate change is a real threat to food and water security in an Arctic health context. However, knowledge on the process of change with regard to food and water security in the Circumpolar area is unknown. The only way to find out which indicators are the most feasible is to demonstrate and use them for comparisons among Arctic countries and to follow the most feasible ones in time. The workshop discussions presented in this article is a humble start of this process. Our wish is that our report will inspire researchers and research funders to act and demonstrate how climate changes affect food and water security, could be best monitored in an Arctic health context. As a first step, a healthy food basket will be used in an international effort to establish a baseline for further studies on food security in the Arctic.
